# The Role of Insulin C-Peptide in the Coevolution Analyses of the Insulin Signaling Pathway: A Hint for Its Functions

**DOI:** 10.1371/journal.pone.0052847

**Published:** 2012-12-27

**Authors:** Shuai Wang, Wei Wei, Yadong Zheng, Junling Hou, Yongxi Dou, Shaohua Zhang, Xuenong Luo, Xuepeng Cai

**Affiliations:** 1 State Key Laboratory of Veterinary Etiological Biology, Key Laboratory of Zoonoses of CAAS, Key Laboratory of Veterinary Parasitology of Gansu Province, Lanzhou Veterinary Research Institute, Chinese Academy of Agricultural Sciences, Lanzhou, Gansu, China; Virgen Macarena University Hospital, Spain

## Abstract

As the linker between the A chain and B chain of proinsulin, C-peptide displays high variability in length and amino acid composition, and has been considered as an inert byproduct of insulin synthesis and processing for many years. Recent studies have suggested that C-peptide can act as a bioactive hormone, exerting various biological effects on the pathophysiology and treatment of diabetes. In this study, we analyzed the coevolution of insulin molecules among vertebrates, aiming at exploring the evolutionary characteristics of insulin molecule, especially the C-peptide. We also calculated the correlations of evolutionary rates between the insulin and the insulin receptor (IR) sequences as well as the domain-domain pairs of the ligand and receptor by the mirrortree method. The results revealed distinctive features of C-peptide in insulin intramolecular coevolution and correlated residue substitutions, which partly supported the idea that C-peptide can act as a bioactive hormone, with significant sequence features, as well as a linker assisting the formation of mature insulin during synthesis. Interestingly, the evolution of C-peptide exerted the highest correlation with that of the insulin receptor and its ligand binding domain (LBD), implying a potential relationship with the insulin signaling pathway.

## Introduction

**Figure 1:**
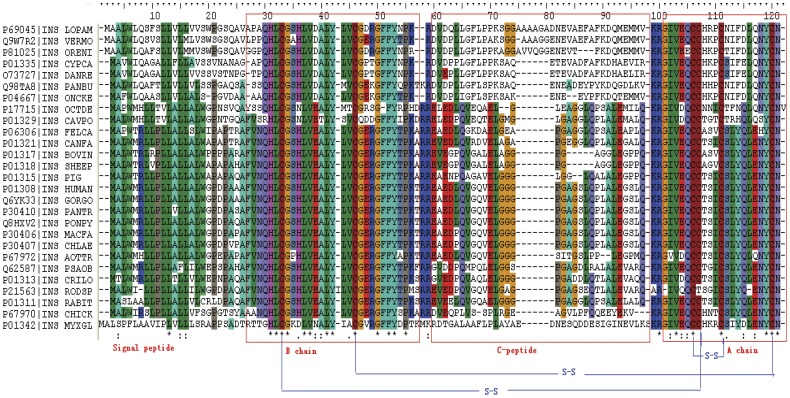
Multiple sequence alignments of insulin protein sequences. The species and Uniprot IDs of insulin sequences are listed in Table S1. The sequence of insulin molecule (preproinsulin) can be divided into signal peptide, B chain, C-peptide and A chain. The latter three constitute the proinsulin, while the B chain and A chain compose the mature insulin with the help of three disulfide bonds.

Insulin is a well-studied neuroendocrine peptide involved in metabolism, growth and survival in a wide range of mammalian tissues. The insulin sequence can be structurally divided into four parts including the signal peptide, B-chain, C-peptide and A-chain (Figure 1). The mature insulin molecule only contains the B chain and A chain linked by three disulphide bonds (Figure 1) and is derived biosynthetically from an insulin precursor, proinsulin which consists of the B and A chains linked to the C-peptide by adjacent pairs of basic residues. However, the initial translation product of the insulin mRNA is preproinsulin, which contains an N-terminal signal peptide linked to proinsulin [1]. The amino acid sequences of the A chain and B chain are highly conserved among vertebrates [2]. However, C-peptide displays high variability in length and amino acid composition (Figure 1). The main role of insulin is to stimulate glucose uptake into cells by inducing the translocation of the glucose transporter GLUT4 from intracellular storage to the plasma membrane [3], [4]. It also functions in glycogen synthesis, DNA replication, fatty acid and protein synthesis and modifications of the activities of numerous enzymes [5].

The C-peptide roles seem negligible because of the cleavage from proinsulin. Actually, C-peptide had been regarded as an inert byproduct of insulin synthesis and processing, only playing an essential role in the insulin synthesis in that it links the A and B chains in a manner that allows correct folding and interchain disulfide bond formation [6]. However, increasing evidence has recently emerged from several laboratories that C-peptide has great potential relevance to the pathophysiology and treatment of diabetes, possibly acting as a peptide hormone beneficially affecting renal, nervous and microvascular functions in diabetic animals [7]–[12].

In the case of C-peptide, the lack of a conserved active site has hampered the recognition of C-peptide as a bioactive hormone [6]. Although, much evidence has demonstrated the presence of the C-peptide receptor, the attempts to identify the exact receptor has failed [7]. Regardless, there can now be no doubt that C-peptide exerts a variety of biological effects. Some sequence features have been identified during investigations of its hormone-like actions and putative receptor. Notably, the residues at positions 1 (Glu) and 6 (Gln) of C-peptide are generally conserved in most mammalian species (Figure 1). Moreover, Ido et al. [13], suggested that the glycine-rich segment in the midportion sequence of C-peptide (positions 13–17) was well conserved and comprised a high proportion of nonpolar amino acids, implying probable active sites for the ligand. Recently, some studies suggested that the C-terminal pentapeptide (EGSLQ in human C-peptide and EVARQ in rat C-peptide) of C-peptide which shows a well-defined secondary structure may have the potential to represent the elusive active site of C-peptide itself [14]–[16]. This evidence suggests the presence of crucial residues (such as the C-terminal pentapeptide, Glu1 and Gln6 in C-peptide) which may contribute to maintaining the functions as a hormone-like peptide during evolution.

Except for the sequence features mentioned above,limited analyses of the peptide characteristics based on computational method have been reported. The term coevolution defines the reciprocal evolutionary change in interacting sites. A change in one locus affects the selection pressure at another locus and this change is reciprocal [17]. For intermolecular coevolution, the physical interaction between interactive partners can lead to linked evolutionary change between the binding partners in order to maintain the effective biological interaction [18]. As a result of the interaction, the correlations between the evolutionary distances of interactive proteins over the whole protein sequence are higher than pairs of non-interactive proteins. For intramolecular coevolution, sites in the three-dimensional structure of a protein can constrain each other's change for proper function [18]. Some sites in a protein structure are more directly influenced with each other to maintain structural integrity and function than others, exerting highly correlated variation during evolution. These sites play a crucial role in keeping a stable, valid three-dimensional structure which is basic for protein's function. Therefore, if there are significant coevolutionary signals among some amino acid residues in the C-peptide, it would be a hint for the presence of its biological functions.

In this study, we utilized bioinformatics methods based on multiple sequence alignments of vertebrate insulin and insulin receptor protein sequences to explore coevolution, as well as correlated residue substitutions of the insulin molecule, with an aim to explore the evolutionary characteristics of insulin molecule, and in particular C-peptide. The correlations of evolutionary distances between the insulin and the insulin receptor were also calculated by the mirrortree method [19]. The results revealed distinctive features of the C-peptide in insulin intramolecular coevolution and correlated residue substitutions. Interestingly, C-peptides exerted the highest correlation to evolutionary distances with the insulin receptor, making it necessary to reconsider the relationship of C-peptide with the insulin signaling pathway.

## Materials and Methods

### Sequence collection and alignment

Sequences for the insulin and IR were collected from the National Center for Biotechnology Information (NCBI) (http://www.ncbi.nlm.nih.gov) using the Blast search program [20] and were also retrieved through searches in the Uniprot database (http://www.uniprot.org/). The selected homologous sequences were all vertebrate species including mammals, birds, amphibians and fishes (Table S1). It is important that one insulin sequence and its IR sequence used for the intermolecular coevolution analysis and the calculations for correlations of evolutionary rate must be selected from the same species (Table 1). Multiple sequence alignments were performed using CLUSTAL W software (version 2.1) [21] and confirmed by PHYLIP [22], and then were manually edited using BioEdit software (version 7.0.9) (http://www.mbio.ncsu.edu/BioEdit/bioedit.html). We performed the multiple sequence alignments with 27 vertebrate insulin protein sequences for covariation and intramolecular coevolution analyses (Table S1). Moreover, the insulin sequences of 12 species together with their corresponding IR sequences (Table 1) were aligned for the intermolecular coevolution analyses and the calculations for correlations of evolutionary rate. Further alignments were all performed using the same software. The human insulin sequence acted as the reference sequence for all the analyses and results described herein.

**Table 1:** The protein sequences for analysis of correlated evolution rates by the mirrortree method.

Species	insulin	IR	CYP17a1
*Homo sapiens*	U: P01308	U: P06213	U:P05093
*Mus musculus*	U: P01325	U: P15208	U: P27786
*Rattus norvegicus*	G: AAA41439.1	U: P15127	U: P11715
*Cavia porcellus*	U: P01329	G: XP_003468418.1	U: Q64410
*Xenopus laevis*	U: P12706	U: Q9PVZ4	U: Q9DDJ5
*Bos taurus*	U: P01317	G: XP_002688878.2	U: P05185
*Canis familiaris*	U: P01321	G: XP_542108.3	U: E2RKV5
*Danio rerio*	U: O73727	G: NP_001136144.1	U: B3DH80
*Gallus gallus*	U: P67970	G: XP_001233399.2	U: P12394
*Oryctolagus cuniculus*	U: P01311	G: XP_002722044.1	U: G1TEG0
*Equus caballus*	G: XP_003362686.1	G: XP_001496634.1	U: Q95328
*Oreochromis niloticus*	U: P81025	G: XP_003448585.1	G: BAF75924.1

The “U” and “G” represent the accession number of protein sequences from UniProtKB/Swiss-Prot database and Genebank database respectively. The species used for correlated evolution rate analysis and intermolecular coevolution analysis were mammals, birds, amphibians and fishes. The gene cytochrome P450, family 17(Cyp17a1) which was not deemed as having direct relation to insulin or IR was chosen as the negative control.

### Covariation analysis of the insulin molecule

The analysis of coordinated substitutions in multiple alignments of protein sequences, which is based on the assumption that functionally coordinated residues in proteins originated by physicochemical properties (e.g., charge, volume, polarity, and hydrophobicity) [23], allows inferences to be made about the structural–functional role of residues at these positions and to predict protein structure [24], [25]. The CRASP (Correlation analysis of protein sequences) program is based on estimation of the correlation coefficient between the values of a physicochemical parameter at a pair of positions of sequence alignment. This enables us to detect and analyze pairwise relationships between amino acid substitutions at specific positions of insulin and then estimate the contributions of the coordinated substitutions to the evolutionary variability of the insulin molecule in integral physicochemical characteristics of the protein residues. The aligned insulin peptide sequences of 27 species were analyzed for pairwise positional correlations to obtain the data of the covariant residues so as to further study the protein evolution. Several calculation parameters of amino acid physicochemical properties (volume, flexibility, polarity, and hydrophobicity) were used for estimating the physical and chemical interactions between residues. All the analysis were at the 99% confidence level and visualized at a clustering cutoff value of 0.55 with other calculation parameters setups acquiescent (the AAindex number is zero, type of matrix is linear correlation and variability threshold value is three). The weighting method suggested by Vingron and Argos [26] was utilized. The calculated matrix was represented as significant pairs with human insulin as the reference sequence. The CRASP program is available at http://wwwmgs.bionet.nsc.ru/mgs/programs/crasp/.

### Intra- and intermolecular coevolution analysis

To identify coevolutionary features of the insulin molecule, we used a parametric method based on correlated changes among amino acid sites [27] implemented in CAPS (Version 1.0) [28]. Coevolution analysis using protein sequences (CAPS) computes the correlated variance of the evolutionary rates at two sites corrected by the divergence time of the protein sequences they belong to [27]. The aligned insulin and IR sequences were analyzed using a number (1000) of random samplings and a threshold alpha-value of 0.05 with the NMR structure of Human proinsulin (PDB ID: 2KQP) as the control file [29]. The minimum R-value to detect a pair of co-evolving sites was 0.1 and the maximum number of sites in a group of co-evolving sites was limited to five percent of the protein length. The preproinsulin 3D structure were predicted by I-TASSER server [30] for revealing the structure relationships between the signal peptide and other parts of preproinsulin. The graphical output for the coevolutionary amino acids was manually visualized by PYMOL(version 1.5) [31]. The coevolutionary networks of amino acids identified by CAPS were visualized by Cytoscape (Version 2.7.0) [32].

### Correlated evolution rate analysis

We utilized the mirrortree method [33] to assess the degree of correlated evolution of interactive proteins [34]. The method is dependent on the observation that the evolutionary distances of interacting proteins often display a higher level of similarity than those of non-interacting proteins [19], [35]–[37]. The preproinsulin, proinsulin and the lone C-peptide sequence together with the whole IR protein sequence, the α-chain and the LBD domain of the IR were all involved in the calculation, with domain ranges annotated at the Uniprot database and confirmed by protein sequence alignments. We also chose the gene cytochrome P450, family 17(Cyp17a1) which was not deemed as having direct relation to above-mentioned proteins as the negative control. Evolutional pairwise distance matrices were constructed by MEGA2 [38] based on the former multiple sequence alignments results using the Poisson model as the substitution method.

The correlation value of each sequence pair was generated by a statistical method based on the distance matrix constructed above. For an X-Y protein or domain pair, the linear correlation coefficient r (Pearson's correlation coefficient) between them was calculated according to the following equation [39]:
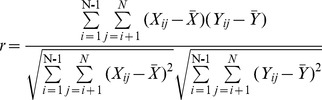
(Equation 1)The X_ij_ or Y_ij_ stands for the evolutionary distance between the i species and j species in the respective sequence matrix of protein (domain) pairs; 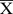 or 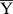 stands for the mean of all X_ij_ or Y_ij_ values, respectively; and N is the species number of the matrices.

## Results

### Covariation analysis

**Figure 2:**
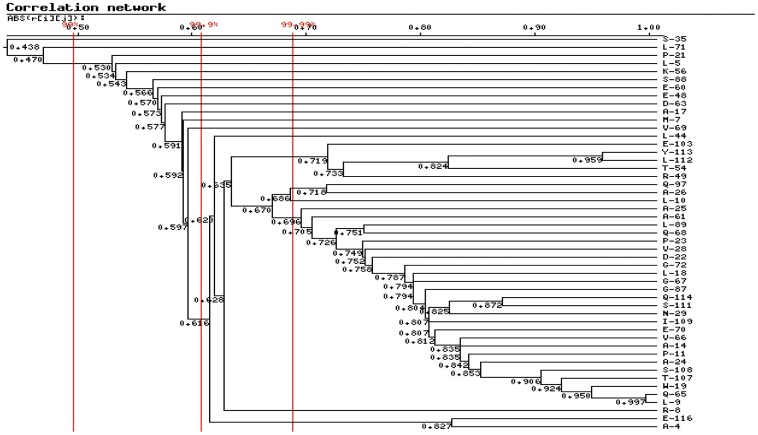
Binary tree diagram for clusters of correlated positions in the insulin molecule. The number below each node indicates the correlation coefficient value. The vertical red bars indicate different significance thresholds. The reference sequence is human insulin.

**Figure 3:**
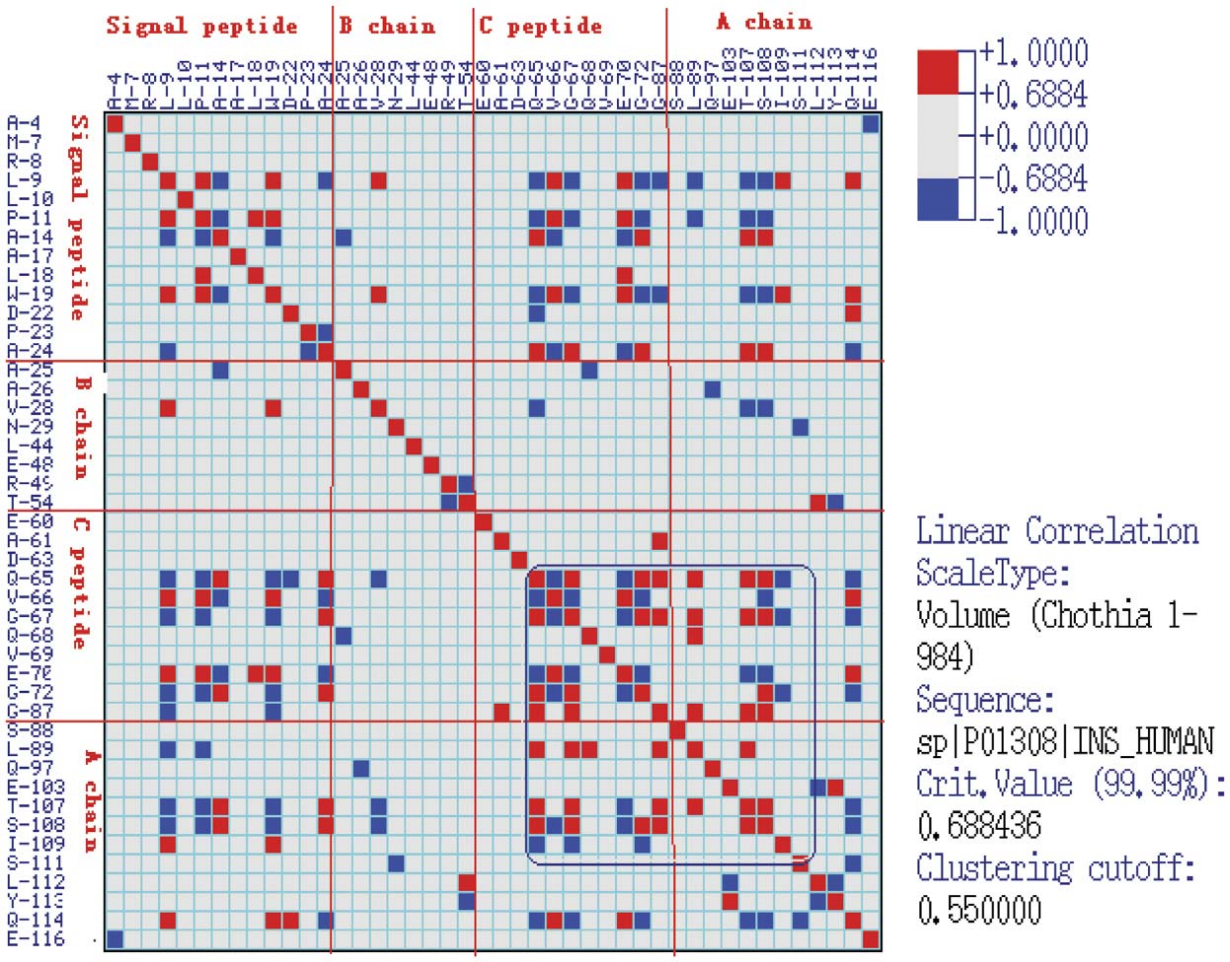
Close view of correlations in significant clusters based on the volume property of amino acids. The residues with significantly correlated substitutions involved in the pairwise dependencies matrix are classified into four columns vertically and horizontally according to the structure of the insulin sequence. The red block diagram represents positive correlation and the blue block diagram represents the negative correlation. Both are at the 99.99% significance level.

As shown in Figure 1, among the species, the A chain and B chain are highly conserved especially in the regions of cysteine residues. Nevertheless, the hormone-like C-peptide sequences among the vertebrates display high variability in length and amino acid composition. A comparison of the amino acid sequences of insulin reveals that, in addition to the six invariant cysteine residues, only a finite number of amino acid residues in the molecule are fully conserved during the evolution of vertebrates. Included in these are the five residues (IleA2, ValA3, TyrA19, GlyB23, and PheB24, numbered in the human insulin sequence) that constitute the proposed receptor-binding domain deduced from the results of alanine-scanning mutagenesis studies [40]. The CRASP program provides the characteristics (such as volume, flexibility, polarity, and hydrophobicity) of amino acids, which reflects the physical and chemical interactions between residues. The covariation result of amino acids characteristics as volumes are shown in Figure 2 and Figure 3. As shown in the horizontal or the vertical column of C-peptide, the amino acid residues of C-peptide has the higher correlated residue substitutions level with the amino acids residues of either itself or the A chain than the other sites (see the blue square frame). The signal peptide show more negative correlations between residue substitutions with other domains (Figure 2 and Figure 3). B-chain has the least number of amino acids of the covariation. The A chain, which has the moderate variability, has the maximum number of covariant residues with the C-peptide. We also examined some other amino acid characteristics (polarity, hydrophobicity, and flexibility) to perform the covariation analyses. Results from the other properties are similar to the result based on volumes characteristic analysis, all of which reveal a significant level of correlated residue substitutions.

### Intra- and intermolecular coevolution analysis

**Figure 4:**
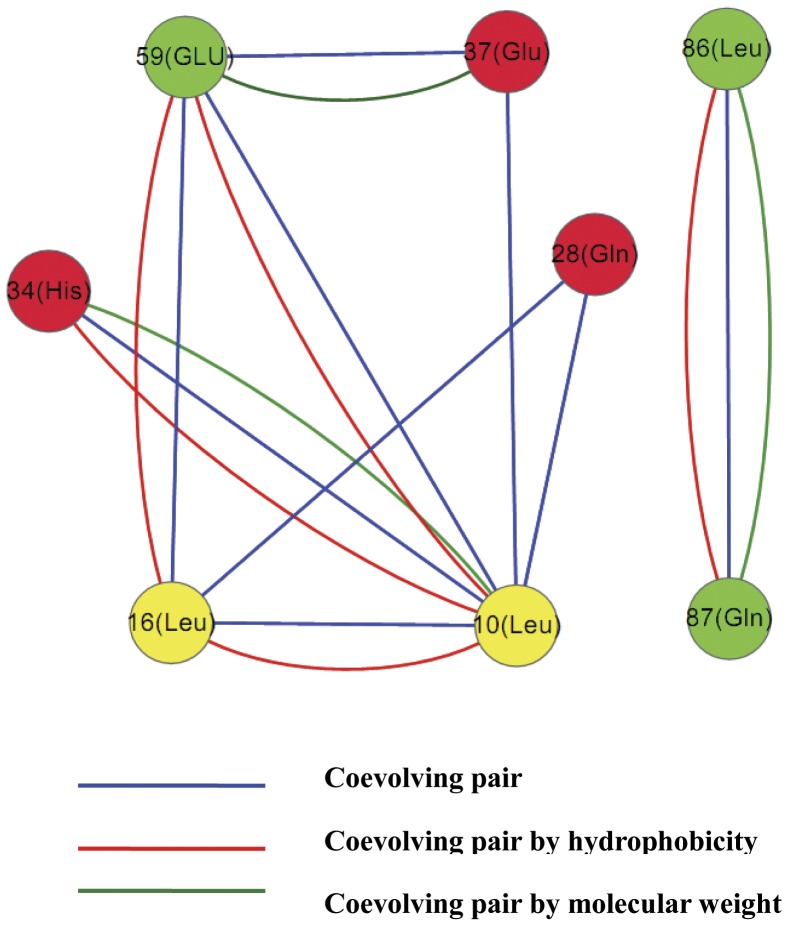
The coevolutionary network of amino acid residues in the insulin molecule. The red nodes represent the amino acid residues from B-chain and the yellow nodes represent the amino acid residues from signal peptide; The green nodes represent amino acid residues from C-peptides.

**Figure 5:**
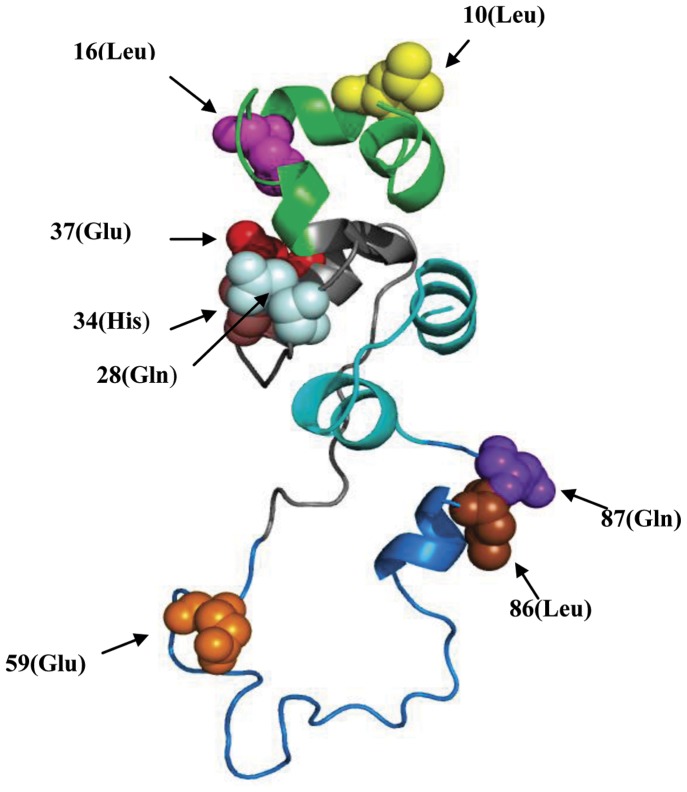
The distribution of intramolecular coevolution amino acid residues mapped onto the human preproinsulin molecule 3D structure. The ribbons with different colors represent the four parts constituting the preproinsulin molecule, appearing as the signal peptide (green), B-chain (gray), C-peptide (skyblue), A-chain (cyan) in order. The amino acids involved in the coevolutionary analysis are displayed in spheres.

The intramolecular coevolution analysis result from the CAPS is listed in Table 2. There are eight groups with clear coevolutionary relevance involving 6 amino acids using the former 27 species alignment for analysis. CAPS also performs a preliminary analysis of compensatory mutations by testing the correlation in the hydrophobicity and the molecular weight variations between coevolving amino acids [28] (Table 2 and Figure 4). The preproinsulin residues 59 (Glu), 86 (Leu) and 87 (Gln) are included in the C-peptides sequence and the 28 (Gln), 34 (His), 37 (Glu) are residues of B-chain, while the 10 (Leu) and 16 (Leu) are the residues from signal peptide. Most of the coevolving residues significantly displayed high correlation coefficients either in hydrophobicities or molecular weights and the coevolution between 34 (His) and 10 (Leu), as well as the coevolution between 86 (Leu) and 87 (GLn) includes both factors. In order to estimate the spatial distance between amino acid residues and probe the contribution of the biophysical chemical properties to the interaction between site pairs, the coevolutionary residues were indicated in a three-dimensional structure of human proinsulin (Figure 5). From the perspective of spatial distance, the residue pairs, 10 (Leu) and 16 (Leu), 10 (Leu) and 28 (Gln), 16 (Leu) and28 (Gln), 10 (Leu)and 34(His), 10 (Leu) and 37 (Glu), 86 (Leu) and 87 (Gln) are within spatial proximity of each other in insulin molecule. The residues of C-peptide involved in the coevolutionary network should link functionally or structurally with their pairs that are subjected to strong selective constraints and evolve together, indicating its essential roles in the function of insulin molecule or the hormone-like function of C-peptide itself during evolution.

**Table 2:** The analysis result of the intramolecular coevolutionary amino acid residues by CAPS.

Site1	Site2	Coevolution correlation	Hydrophobicities correlation	Molecular weights correlation
10	16	0.9421	0.8974	No
10	28	0.9784	No	No
16	28	0.9239	No	No
10	34	0.8726	0.8098	0.8863
10	37	0.8688	No	No
10	59	0.9637	0.6633	No
37	59	0.9202	No	0.4980
16	59	0.9294	0.6858	No
86	87	0.9822	0.9900	0.9900

“No” indicates no significant correlation was detected.

The intermolecular coevolution analyses by CAPS between insulin and IR with the 12 species (Table 1) failed to detect the coevolutionary residues between the protein pairs perhaps. The intramolecular coevolution network is shown in Figure 4.

### Correlated evolution rates analysis

**Figure 6:**
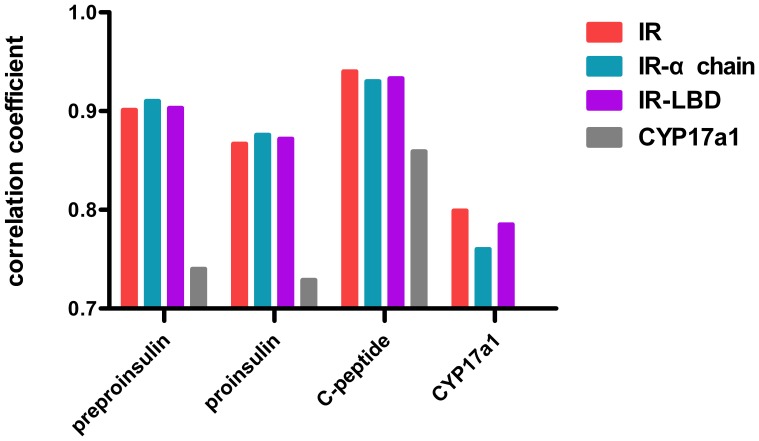
The refined evolutionary correlations between the ligand and receptor. The CYP17a1 protein sequences are calculated as the control, pairwise with all other sequences. The C-peptide shows the maximum correlation value level. Each pair with the control is lower than the positive pair in every group.

Dou et al. [39] have used the mirrortree method to calculate the evolutionary correlations of insulin/insulin like growth factor I signal pathway in vertebrate species. Their data showed that the ligands share high correlation coefficient values with their receptors. Our results of correlations between different domain pairs can be seen in Figure 6. For each group, the control value is lower than the positive pair, demonstrating that the results reflecting the coevolution level are valid. As is shown in Table 3, all groups of the ligand share high correlation values with the receptor domains [33]. Surprisingly, the correlated evolution between C-peptide and the receptor or receptor ligand binding domains presents the highest correlation values compared with the other groups.

**Table 3:** The evolutionary correlation values between the ligand and receptor.

	preproinsulin	proinsulin	C-peptide	CYP17a1(control protein)
IR	0.901	0.867	0.940	0.799
IR-α chain	0.910	0.876	0.930	0.760
IR-LBD	0.903	0.872	0.933	0.785
Mean	0.905	0.872	0.934	0.781
CYP17a1	0.740	0.729	0.859	1

As supported by the coevolution rationale [36], [37], [39], [41] demonstrated by many studies which indicate that interacting protein pairs exhibit higher level of correlated evolution than the non-interacting protein pairs [34], the preproinsulin or proinsulin sequence group shows a higher correlated evolution level than the control groups or the artificial false pairs.

## Discussion

The differences in molecules characteristics among the parts of the preproinsulin molecule may result in the diversity of coevolutionary and covariant features. The fact that the B-chain has the least number of the covariant sites based on the result of the CRASP analysis may be reasonably explained by the relatively high conservation of its sequence, while it has three coevolutionary sites, 28 (Gln), 34 (His) and 37 (Glu) which may affect its binding affinity to the insulin receptor [40]. This can be supported by the fact that the A chain sequence exerts moderate conservation compared with the B chain and C-peptide and it shows the medium number of correlated sites of residue substitutions. From an overall perspective, the covariant connections and correlated evolution between insulin residues reflect the strong physical and chemical interactions between them that generate strong selective constraints. Based on the studies of three-dimensional structure interactions for insulin and its receptor, the A chain primarily interacts electrostatically with the L1 and L2 domains of one IR-α subunit with no obvious hydrophobic components while the B chain interacts with the other subunit, chiefly hydrophobically with the L1 domain and electrostatically with the CR region [42]. This can partly explain the result of the intramolecular coevolution analysis (Table 2 and Figure 4) where the coevolutionary residues involving the B chain are chiefly discovered in the hydrophobicity factor. This different interaction pattern might explain the difference in the variability, covariation and coevolution level between the A chain and B chain, because the residue substitutions resulting in changes of characteristics such as hydrophobic and electrostatic dynamics can impose strong restrictions on protein sequence evolution [18].

The C-peptide displays high correlations between residue substitutions and has significant levels of compensatory mutations at 59 (Glu), 86 (Leu) and 87(Gln) in hydrophobicities and molecular weights. Hydrophobicities and molecular weights are among the most important factors in explaining amino acid contribution to protein structure with less error [43]. The significant coevolution characteristic of C-peptide may imply the crucial role in the insulin molecule, although it displays high variability in length and amino acid composition. This is supported by the result from mutant stability changes analysis that most of the mutations to alanines for the residues involved in the coevolution analysis by CAPS would destabilize the structure of proinsulin which may impair the protein folding (Text S1, Table S2). During the course of insulin synthesis, the C-peptide is essential for the correct folding and interchain disulfide bonds formation of the mature insulin. When C-peptide is removed from proinsulin by proteolytic processing, the COOH-terminal part of insulin's B-chain becomes exposed and is free to assume an appropriate conformation for effective interaction with the insulin receptor. The obvious signatures of correlated variance in C-peptide represent the relatively functional conservation during the evolution. The coevolutionary residue 59 (Glu) of C-peptide shares the distant interaction in space with 37 (Glu) in the B chain for a significant coevolution correlation coefficient either in hydrophobicities or molecular weights. This may be explained by the contribution of C-peptide to the formation of the correct conformation for the mature insulin molecule binding to the IR. And this is supported by the observation that alanine scanning mutagenesis or deletion of 59 (Glu) at the N-terminus of the C-peptide in human proinsulin resulted in serious aggregation during refolding, which could imply a crucial role of the highly conserved acidic residue for insulin precursor folding [44], [45]. From the hormone-like peptide point of view, the high level of correlation of residues substitutions correlation within C-peptide sequence and the closely related coevolution sites with the mature insulin chains may reverse the constraint force for the C-peptide function and evolution.

Clearly if C-peptide can function as a hormone-like peptide, it most probably affirms the existence of a receptor. In the classic ligand- receptor interaction manner, there must be a limited region of the ligand serving as “active sites”, affecting the binding to the receptor, which is generally well conserved across species borders [6]. Although the attempt to identify the exact C-peptide receptor has failed, binding assays using new technology have established a typical receptor interaction for C-peptide [46]. Consequently, it now appears that C-peptide can be recognized as a receptor ligand with no doubt; it is just that some properties were difficult to define initially and that additional or multiple effects may exist [6]. Several mechanisms have been put forward to explain the molecular interactions between C-peptide and its receptor [6]. In our results, we found that the C-peptide had a significant role in the coevolution and had significant sequence features during evolution, which corroborates the claim that C-peptide can act as a hormone-like cytokine which has important functions in the pathophysiology and treatment of diabetes, and that it may evolve in a manner more like a biological factor with significant correlation and restriction of residues mutation. Moreover, Ido et al. [13] suggested that the midportion sequence of C-peptide, largely conserved, and comprising a high proportion of nonpolar amino acids was implicated in the ligand activity. In addition, the coevolutionary residues 86 (Leu) and 87 (Gln) of human proinsulin in the C-terminal pentapeptide of C-peptide may be involved in the elusive active sites of the C-peptide itself and participate in the interaction with its specific receptor [14]–[16]. Therefore, the relatively conserved region and the residues with notably correlated variability may construct the basis of the ligand concept.

Intermolecular coevolution analysis between insulin and IR by the CAPS with the 12 species failed to detect the coevolutionary residues between the protein pairs. This failure is most likely ascribed to the limited number of homologous sequences in alignment which can negatively affect the sensitivity of the method for detecting coevolution [27].

Based on the evolutionary distances, the assessment of the agreement between the evolutionary histories of two proteins or domains was possible. As is shown in Table 3 and Figure 5, preproinsulin and proinsulin both share a very high correlation with the IR or the ligand binding domain of IR. It is a matter of course, when we consider the interaction between the ligand and receptor as well as the analogous evolutionary pressure of the insulin signal pathway [18], [39], [47]. However, the cases of C-peptide seem strange for the not logical coevolutionary correlation. Kann et al. [37] reported that the binding site sequence was subject to stronger correlated evolution than other regions of the interacting protein domains and Raja et al. [34], found that interacting domain pair(s) for a given interaction exhibits higher level of correlated evolution than the noninteracting domain pairs. Both of these observations are in conformity with the principle of the general coevolution theory that proteins and their interactive partners would share correlated evolution and that any divergent changes in one partner's binding surface or interactive domain are complemented at apposite sites by their interaction partners for maintaining the proper binding [37]. But our results suggest that the C-peptide which would be cleaved from the proinsulin during the insulin synthesis and processing displays the highest evolutionary correlation with either IR or LBD among all the domain pairs. This phenomenon may imply that the hormone-like peptide may have a further relationship with the insulin signaling pathway. And several reports have described the ability of C-peptide to induce phosphorylation and activation of members of the MAPK family which are evolutionary conserved enzymes that link cell-surface receptors (including IR) to key regulatory targets within cells [48], [49]. Indeed, Grunberger et al. [50], wondered whether C-peptide signaling may simply be explained by activation of the insulin receptor signaling system. It remains unclear what the identity of C-peptide receptor as well as the relationship between C-peptide and the insulin signal pathway is [6], [7]. Our results reveal the high evolution correlation between C-peptide and the insulin receptor, but do not provide structural evidence of the direct binding. Accumulating evidence shows that the activation may result from other pathways such as a specific GTP-binding protein-coupled receptor pathway or the non-specific binding of C-peptide to the cellular membrane [13], [51]–[53]. So the result may be explained by that C-peptide is closely associated with the insulin signal pathway in a conserved mechanism such as a specific GTP-binding protein-coupled receptor pathway. However, since the mirrortree method is based on common variation of evolution distances between sequences of interacting proteins or domains rather than on a direct measurement of the factors that might contribute to this variation, the exact coevolutionary factors that lead to the highest level of correlated evolutionary distance between C-peptide and IR can not be uncovered. The exact relationship between C-peptide and insulin signaling pathway may be clarified when the expectedly detailed identification/cloning of a receptor is obtained.

In conclusion, C-peptide exerts distinguished features in residue correlated substitutions and compensatory mutations of the insulin molecule during evolution, supporting its role as the helper for mature insulin formation or as a hormone-like peptide in spite of the high variability in length and amino acid composition. Furthermore, the surprisingly highest correlation value of the evolutionary distances with the IR or LBD as well as the significant contribution of C-peptide in correlation between the insulin and its receptor enlightens us on its putative role in the evolution of insulin signal pathway, giving us inspiration that the hormone-like peptide may have a further relationship with the insulin signaling pathway in addition to its role as a linker for proinsulin. All above can help us understand the “double-face” role of C-peptide appearing as a linker and a putative hormone.

## Supporting Information

Text S1 **Prediction of protein mutant stability changes.**
(DOC)

Table S1 Orthologous sequences of insulin and their accession numbers obtained from UniProtKB/Swiss-Prot database.
(DOC)

Table S2 Prediction of protein mutant stability changes for the coevolving sites.
(DOC)
