# Supplementary material for: The Role of Insulin C-Peptide in the Coevolution Analyses of the Insulin Signaling Pathway: A Hint for Its Functions
Source: PLoS One. 2012 Dec 27;7(12):e52847. doi: 10.1371/journal.pone.0052847 (PMC3531361; doi:10.1371/journal.pone.0052847)
Supplement: Text S1 — Prediction of protein mutant stability changes. (DOC) [file pone.0052847.s001.doc]

**Materials and Methods**

**Prediction of protein mutant stability changes**

In order to estimate the possible roles of the coevolving residues involved in the coevolution analysis by CAPS in the synthesis and processing of insulin, predictions of protein mutant stability changes were performed by the PoPMuSiC program (version 2.1). The PoPMuSiC program is a powerful tool to evaluate the changes in stability of a given protein or peptide under single-site mutations, on the basis of the protein's structure. The mutant stabilities of alanine substitutions in positions from the CAPS analysis were evaluated in preproinsulin based on the 3D structure of proinsulin (2KQP). Other control parameters were set the default. The solvent accessibility, the change in folding free energy upon mutation (ΔΔG) and stability change were all involved in the analysis.

**Results**

**Prediction of protein mutant stability changes**

The coevolving sites 10, 16, 28, 34, 37, 59, 86 and 87 of preproinsulin involved in the CAPS analysis were mutated to alanines for the purpose of predicting the protein mutant stability changes. The result showed that the most of the mutations to alanines given positive ΔΔG values which would destabilize the structure of the protein (Table S2). The mutation of site 10, 16, 28, 34, 59 and 86 led to a destabilizing effect on the preproinsulin structure with positive ΔΔG values while the mutations of sites 37 and 87 exerted negative ΔΔG values near zero.

References

1. Dehouck Y, Kwasigroch JM, Gilis D, Rooman M (2011) PoPMuSiC 2.1: a web server for the estimation of protein stability changes upon mutation and sequence optimality. BMC Bioinformatics 12: 151.
