# Supplementary material for: The Role of Insulin C-Peptide in the Coevolution Analyses of the Insulin Signaling Pathway: A Hint for Its Functions
Source: PLoS One. 2012 Dec 27;7(12):e52847. doi: 10.1371/journal.pone.0052847 (PMC3531361; doi:10.1371/journal.pone.0052847)
Supplement: Table S1 — Orthologous sequences of insulin and their accession numbers obtained from UniProtKB/Swiss-Prot database. (DOC) [file pone.0052847.s002.doc]

**Table S1.** Orthologous sequences of insulin and their accession numbers obtained from UniProtKB/Swiss-Prot database

| **Species** | **Accession number** | **Species** | **Accession Number** |
| --- | --- | --- | --- |
| [*Lophius americanus*](http://www.uniprot.org/taxonomy/8073) | **P69045** | [*Homo sapiens*](http://www.uniprot.org/taxonomy/9606) | **P01308** |
| [*Verasper moseri*](http://www.uniprot.org/taxonomy/98923) | **Q9W7R2** | ***Gorilla gorilla*** | **Q6YK33** |
| [*Oreochromis niloticus*](http://www.uniprot.org/taxonomy/8128) | **P81025** | [*Pan troglodytes*](http://www.uniprot.org/taxonomy/9598) | **P30410** |
| [*Cyprinus carpio*](http://www.uniprot.org/taxonomy/7962) | **P01335** | [*Pongo pygmaeus*](http://www.uniprot.org/taxonomy/9600) | **Q8HXV2** |
| [*Danio rerio*](http://www.uniprot.org/taxonomy/7955) | **O73727** | [*Macaca fascicularis*](http://www.uniprot.org/taxonomy/9541) | **P30406** |
| [*Pantodon buchholzi*](http://www.uniprot.org/taxonomy/8276) | **Q98TA8** | [*Chlorocebus aethiops*](http://www.uniprot.org/taxonomy/9534) | **P30407** |
| [*Oncorhynchus keta*](http://www.uniprot.org/taxonomy/8018) | **P04667** | [*Aotus trivirgatus*](http://www.uniprot.org/taxonomy/9505) | **P67972** |
| [*Octodon degus*](http://www.uniprot.org/taxonomy/10160) | **P17715** | [*Psammomys obesus*](http://www.uniprot.org/taxonomy/48139) | **Q62587** |
| [*Cavia porcellus*](http://www.uniprot.org/taxonomy/10141) | **P01329** | [*Cricetulus longicaudatus*](http://www.uniprot.org/taxonomy/10030) | **P01313** |
| [*Felis catus*](http://www.uniprot.org/taxonomy/9685) | **P06306** | [*Rodentia sp.*](http://www.uniprot.org/taxonomy/69158) | **P21563** |
| [*Canis familiaris*](http://www.uniprot.org/taxonomy/9615) | **P01321** | [*Oryctolagus cuniculus*](http://www.uniprot.org/taxonomy/9986) | **P01311** |
| [*Bos taurus*](http://www.uniprot.org/taxonomy/9913) | **P01317** | [*Gallus gallus*](http://www.uniprot.org/taxonomy/9031) | **P67970** |
| [*Ovis aries*](http://www.uniprot.org/taxonomy/9940) | **P01318** | [*Myxine glutinosa*](http://www.uniprot.org/taxonomy/7769) | **P01342** |
| [*Sus scrofa*](http://www.uniprot.org/taxonomy/9823) | **P01315** |  |  |
