# Supplementary material for: The Role of Insulin C-Peptide in the Coevolution Analyses of the Insulin Signaling Pathway: A Hint for Its Functions
Source: PLoS One. 2012 Dec 27;7(12):e52847. doi: 10.1371/journal.pone.0052847 (PMC3531361; doi:10.1371/journal.pone.0052847)
Supplement: Table S2 — Prediction of protein mutant stability changes for the coevolving sites. (DOC) [file pone.0052847.s003.doc]

**Table S2.** Prediction of protein mutant stability changes for the coevolving sites

| **Site** | mutations | **Solvent accessibility** | **ΔΔG** | **stability** |
| --- | --- | --- | --- | --- |
| 10 | Leu to Ala | 53.34% | 0.45 kcal/mol | destabilizing |
| 16 | Leu to Ala | 36.77% | 1.46 kcal/mol | destabilizing |
| 28 | Gln to Ala | 42.96% | 0.65 kcal/mol | destabilizing |
| 34 | His to Ala | 28.15% | 0.63 kcal/mol | destabilizing |
| 37 | Glu to Ala | 48.87% | -0.22 kcal/mol | stabilizing |
| 59 | Glu to Ala | 68.74% | 0.04 kcal/mol | destabilizing |
| 86 | Leu to Ala | 31.59% | 1.32 kcal/mol | destabilizing |
| 87 | Gln to Ala | 100.00% | -0.06 kcal/mol | stabilizing |
